# Mode of birth in subsequent pregnancy when first birth was vacuum extraction or second stage cesarean section at a tertiary referral hospital in Uganda

**DOI:** 10.1186/s12884-024-06282-9

**Published:** 2024-02-01

**Authors:** Assen Kamwesigye, Barbara Nolens, Herbert Kayiga, Moses Muriuki, Wani Muzeyi, Jolly Beyeza-Kashesya

**Affiliations:** 1 Department of Obstetrics and Gynecology, Mbale Regional Referral Hospital, Mbale, P.O. Box 921, Uganda; 2 Canisius-Wilhelmina Hospital, Nijmegen, the Netherlands; 3 Department of Obstetrics and Gynecology, Makerere University College of Health Sciences, Kampala, Uganda; 4 Makerere University College of Health Sciences, Kampala, Uganda

**Keywords:** Assisted vaginal birth, Cesarean section, Maternal outcome, Mode of birth, Neonatal outcome, Vacuum extraction

## Abstract

**Introduction:**

The trends of increasing use of cesarean section (CS) with a decrease in assisted vaginal birth (vacuum extraction or forceps) is a major concern in health care systems all over the world, particularly in low-resource settings. Studies show that a first birth by CS is associated with an increased risk of repeat CS in subsequent births. In addition, CS compared to assisted vaginal birth (AVB), attracts higher health service costs. Resource-constrained countries have low rates of AVB compared to high-income countries. The aim of this study was to compare mode of birth in the subsequent pregnancy among women who previously gave birth by vacuum extraction or second stage CS in their first pregnancy at Mulago National Referral Hospital, Uganda.

**Methods:**

This was a retrospective cohort study that involved interviews of 81 mothers who had a vacuum extraction or second stage CS in their first pregnancy at Mulago hospital between November 2014 to July 2015. Mode of birth in the subsequent pregnancy was compared using Chi-2 square test and a Fisher’s exact test with a 0.05 level of statistical significance.

**Results:**

Higher rates of vaginal birth were achieved among women who had a vacuum extraction (78.4%) compared to those who had a second stage CS in their first pregnancy (38.6%), *p* < 0.001.

**Conclusions and recommendations:**

Vacuum extraction increases a woman’s chance of having a subsequent spontaneous vaginal birth compared to second stage CS. Health professionals need to continue to offer choice of vacuum extraction in the second stage of labor among laboring women that fulfill its indication. This will help curb the up-surging rates of CS.

## Introduction

The increasing trends in cesarean section (CS) with a decrease in assisted vaginal birth (AVB) such as vacuum extraction or forceps aided birth is a major concern in health care systems all over the world, particularly in low-resource settings [1]. The management of the first time mother with a singleton cephalic pregnancy at term seems to be a major contributor to the increase in rates of CS [2]. Whereas a CS can be a life-saving intervention when medically indicated, the procedure is an important determinant of mode of birth in subsequent pregnancies. High rates of spontaneous vaginal births can be achieved after a previous AVB [3]. This is not the case after a previous CS. In many settings, a previous CS is one of the commonest predictors of a subsequent CS [4]. Moreover, CS can have detrimental effects on the health of mother and baby, especially in our low-resource setting which faces resource restrictions and where access to care, especially access to theatre, in the subsequent pregnancy is not guaranteed [5].

Prevention of unnecessary second stage CS by use of evidence based interventions such as AVB could help mitigate the rising CS rates [6]. A study conducted between November 2014 and July 2015 at Mulago Hospital that compared maternal and neonatal outcomes after vacuum extraction and second stage CS revealed that vacuum extraction was associated with a lower risk of infection and hemorrhage, a shorter decision to birth interval and therefore lower rates of birth asphyxia, intrapartum stillbirths, and severe maternal morbidity [7]. Out of this this original cohort of women, we have surveyed those that were primigravida at that time, from both the vacuum extraction group and the second stage CS group to compare outcomes in the subsequent pregnancy, with mode of birth being the primary outcome of our study.

## Methods

This was a retrospective cohort study with patient reported outcomes. Mode of birth and other outcomes of the second pregnancy were compared between two groups. One group consisted of mothers who had vacuum extraction in their first pregnancy, the other group consisted of mothers who gave birth by second stage CS in their first pregnancy.

The study was conducted in February and March 2020 from the out-patients’ department at Kawempe National Referral Hospital (KNRH), a public facility in Kampala, Central Uganda. KNRH currently houses the department of Obstetrics and Gynecology of Mulago hospital.

The study was a 5-year follow-up study of a study in Mulago hospital between November 2014 and July 2015 at Mulago Hospital that sought to compare maternal and neonatal outcomes after vacuum extraction and second stage CS. Contact details of the primiparous mothers were retrieved from the database. The mothers had consented for a follow up study prior to their discharge from hospital in 2014–2015.

The assumption was, that many of these mothers had already had their subsequent birth. We contacted the mothers using their telephone contacts. Data was collected using an interviewer administered structured questionnaire. Questions were asked about the first ongoing pregnancy (abortions were not analyzed) that followed the woman’s first pregnancy in which she had a vacuum extraction or second stage CS in 2014–2015. In other words, the pregnancy that made her para two.

### Data management and statistical analysis

The collected data was coded and double entered into EPIDATA to ensure validation. The data was then exported to STATA version 14.0 for analysis. Participants’ baseline characteristics were presented in form of frequencies and percentages.

Subsequent mode of birth was compared between participants who had a previous vacuum extraction and those who had a second stage CS using Chi-2 square test and a Fisher’s exact test with a 0.05 level of statistical significance.

## Results

**Fig. 1:**
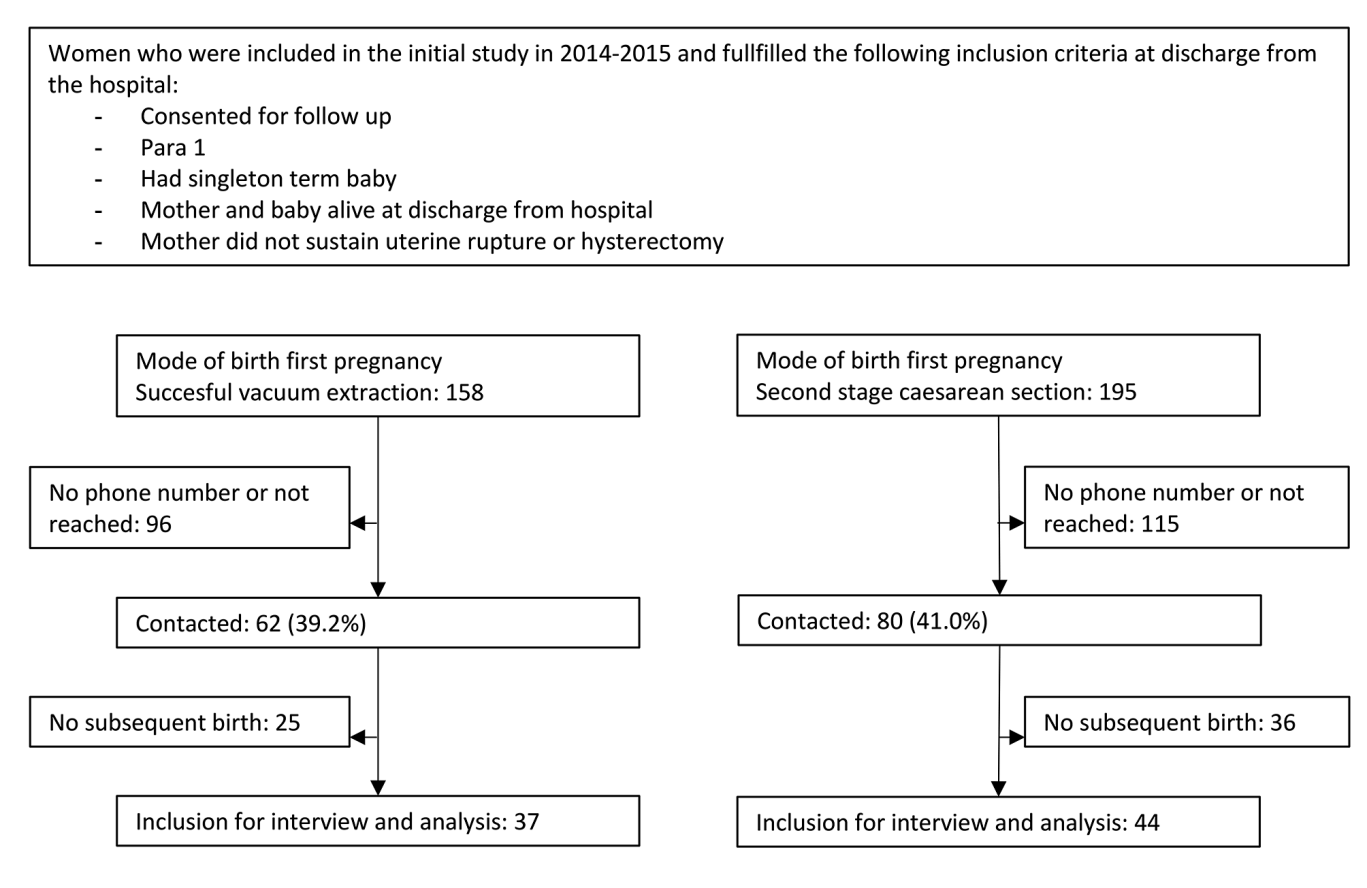
Inclusion process

We were able to contact 142 (40.2%) out of the 353 primigravidas enrolled in the primary study and eligible for the current study [7]. Follow up rates were similar for both groups (39.2% for group with previous vacuum extraction and 41.0% for group with previous second stage CS). Of the contacted women, 81(57.0%) had had a subsequent birth and they were all included in our study (Fig. 1).

Table 1 (demographic characteristics) shows that majority (80.2%) of the participants were less than 30 years of age with a mean age of 27.4 and 60.5% had attained secondary level of education. Nearly a half of the participants had informal employment. Majority (92.5%) of the participants lived within a distance of six kilometers from a public health facility.

**Table 1:** Social and demographic characteristics of the study participants

Variable	Frequency (*N* = 81)	Percent (%)
**Age**		
< 30	65	80.2
≥30	16	19.8
**Education**		
primary	15	18.5
secondary	49	60.5
Tertiary	17	21.0
**Occupation**		
Unemployed	32	39.5
Formally employed	12	14.8
Informal employment	37	45.7
**Distance form HC**		
< 6 km	75	92.6
≥ 6 km	6	7.4
**Interview type**		
Physical	50	61.7
Phone	31	38.3
**Reasons for Phone interview**		
Far	12	38.7
Busy	14	45.2
Other	5	16.1

Table 2 (obstetric characteristics) shows that there were 37 mothers with previous vacuum extraction and 44 mothers with previous second stage CS included in our study. There were no statistically significant differences between the groups. The majority (93.8%) of the mothers carried their pregnancy to term. One mother in the previous vacuum extraction group had a preterm birth in the following pregnancy (1/37, 2.6%) compared to four mothers in the previous CS group (4/44, 9.1%). This was not statistically significant.

**Table 2:** Obstetric characteristics of the study participants in the subsequent pregnancy in relation to the mode of birth in the first pregnancy

Variable	Vacuum extraction in first pregnancy (*n* = 37) n(%)	Second stage cesarean section in first pregnancy (*n* = 44) n(%)	***P*** value
**Inter pregnancy interval**			0.079
< 24 Months	12(32.4)	22(50.0)	
≥24 Months	25(67.6)	22(50.0)	
**Medical illness in subsequent pregnancy**			0.227
Hypertension	3(8.1)	1(2.3)	
No medical illness	34(91.9)	43(97.7)	
**Pregnancy intention**			0.570
Planned	17(45.9)	23(52.3)	
Unplanned	20(54.1)	21(47.7)	
**Gestation age at subsequent birth**			0.379
Preterm	1(2.7)	4(9.1)	
Term	36(97.3)	40(90.9)	
**ANC attendance**			0.662
Yes	36(97.3)	42(95.5)	
No	1(2.7)	2(4.5)	

Table 3 shows maternal outcomes in the subsequent pregnancy. Of mothers that had a previous vacuum extraction, 29/37 (78.4%) had a subsequent vaginal birth, compared to 17/44 (38.6%) of those who had a previous second stage CS. This difference was significant with a *p* value of < 0.001. In both groups the preferred mode of birth was vaginal birth, 35/37 (94.6%) in the previous vacuum extraction group and 39/44 (88.6%) in the previous second stage CS group. In both groups there were very few elective CS. If mothers had CS in the second pregnancy it was more often an emergency CS: 7/8, (87.5%) in the group who had vacuum extraction in the first pregnancy and 23/27 (85.2%) in the group who had a previous CS.

**Table 3:** Maternal outcomes of the study participants in their subsequent birth in relation to the mode of birth in the first pregnancy

Variable	Vacuum extraction in first pregnancy (*n* = 37) n(%)	Second stage cesarean section in first pregnancy (*n* = 44) n(%)	***P*** value
**Place of birth**			0.111
Public Hospital	23(62.2)	37(84.1)	
Private hospital	12(32.4)	7(15.9)	
Traditional birth attendant/Home birth	2(5.4)	0	
**Mode of birth in subsequent pregnancy**			< 0.001
Vaginal birth	29(78.4)	17(38.6)	
Cesarean section	8(21.6)	27(61.4)	
**Type of cesarean section**			0.869
Emergency	7(87.5)	23(85.2)	
Elective	1(12.5)	4(14.8)	
**Preferred mode of birth**			0.347
Vaginal birth	35(94.6)	39(88.6)	
Cesarean section	2(5.4)	5(11.4)	
**Hospital stay**			< 0.001
1 or 2 days	27(73.0)	13(29.5)	
3 days or more	9(24.3)	30(68.2)	
unknown	1(2.7)	1(2.3)	
**Post-delivery infection**			0.901
Yes	1(2.7)	1(2.3)	
No	36(97.3)	43(97.7)	

Hospital stay for three or more days after the second birth was 24.3% in the group with vacuum extraction in the first pregnancy and 68.2% in the group with second stage CS in first pregnancy (*p* < 0.001). This was mainly caused by longer hospital stay after (repeat) CS in the group of mothers with a second stage CS in their first pregnancy.

Table 4 (neonatal outcomes in the subsequent pregnancy) shows that 33/37 (89.2%) neonates in the previous vacuum extraction group had a birthweight of 2.5 kg or more, compared to 34/44 (77.3%) in the previous CS group. Neonates of mothers with previous CS needed more often admission to the neonatology unit (18.2%, compared to 5.4% in the previous vacuum extraction group) and were more often premature (9.1%, compared to 2.7% in the previous vacuum extraction group). Two mothers from the previous vacuum extraction group gave birth to stillborn in their subsequent births, both through vaginal birth. Of these two mothers, one had suffered severe hypertension requiring termination of pregnancy at seven months of gestation which could have resulted in the fetal death due to prematurity/placental malfunction. The second mother reported loss of fetal movements at term and intrauterine fetal demise was diagnosed, although there were no medical conditions identified that could have resulted in the fetal demise.

**Table 4:** Immediate neonatal outcomes of the subsequent birth in relation to the mode of birth in the first pregnancy

Variable	Vacuum extraction in first pregnancy (*n* = 37) n(%)	Second stage cesarean section in first pregnancy (*n* = 44) n(%)	***P*** value
***Birth weight***			0.158
< 2.5Kg	4(10.8)	10(22.7)	
≥2.5Kg	33(89.2)	34(77.3)	
**Sex of baby**			0.110
Male	20(54.1)	16(36.4)	
Female	17(45.9)	28(63.6)	
**Status of baby**			0.118
Alive	35(94.6)	44(100)	
Not Alive	2(5.4)	0(0)	
**Admission to neonatology unit**			0.082
Yes	2(5.4)	8(18.2)	
No	35(94.6)	36(81.8)	

## Discussion

### Mode of birth in subsequent pregnancy when first birth was by vacuum extraction or second stage CS

Higher rates of vaginal birth were achieved among women who had a vacuum extraction compared to those that had a second stage CS in their first pregnancy. Similar findings have been reported in a study from Bristol, UK [3] and from Cameroon [8]. This is important because increasing use of CS rather than AVB for the management of poor progress in the second stage of labor in the first pregnancy will have far reaching consequences in following pregnancies. Most women who have had a CS in their first pregnancy have repeat CS in subsequent pregnancies [9]. This increases the overall rate of CS [10] and has a negative effect on maternal health and health costs. Reasons for repeat CS are policies in some maternity centers where “once a scar always a scar” is the rule, fear of uterine rupture, mother’s consent [10], among others. An interesting finding in our study is however that most women with a previous CS had trial of labor and only 4/27 (14.8%) had elective repeat CS. This might reflect the preferred mode of birth of most women; 39/44 (88.6%) in the previous CS group preferred vaginal birth.

Emphasis should be placed on achieving a vaginal birth in the first pregnancy. This will prevent repeat CS and probably prevent complications from repeat CS (post-partum hemorrhage, infection, placenta previa/accreta) [11]. Women who have had an AVB should be reassured by the very high likelihood of achieving a spontaneous vaginal birth in subsequent pregnancies.

### More preterm births in previous CS group

Four mothers (4/44, 9.1%) from the second stage CS group had preterm births in their subsequent pregnancy compared to one (1/37, 2.7%) from the vacuum extraction group. This is not a statistically significant difference (groups too small, study not powered for this outcome). However, the trend is comparable to outcomes of a systematic review and meta-analysis that focused on the relationship between mode of birth (CS vs. vaginal birth) in the first pregnancy and the risk of subsequent preterm birth. The meta-analysis showed that compared with vaginal birth in the first pregnancy, CS in the first pregnancy increases the risk of preterm birth in subsequent pregnancies [12], with second stage CS being an independent risk factor for subsequent preterm birth in another study [13]. It is hypothesized that the uterine structure and/or intrauterine microenvironment may be changed by previous CS, which increases the risk of subsequent preterm birth in the next pregnancies. Cervical trauma in the second stage of labor or unintentional incision into the uterine cervix during the previous CS could also disrupt the cervical integrity. This damage can affect the function of the cervix, and further increase the risk of preterm birth in future pregnancies. In a large retrospective cohort study that compared first birth by second stage CS with AVB for the risk of preterm birth in the subsequent pregnancy, it was found that second stage CS is associated with a significantly higher rate of preterm birth in the subsequent pregnancy compared to AVB [14].

### Preferred mode of birth

Nearly all mothers preferred vaginal birth above CS. Several studies conducted to ascertain women’s preferred mode of birth showed that most women prefer to have a vaginal birth over a CS, mainly because of its presumed safety, being the natural way of giving birth, less cost compared to CS, and social and cultural influence [15, 16]. In one study, the main reason for women to prefer CS was because of (presumed) medical indication or because of doctors’ remarks [16].

Integration of women’s preferred mode of birth into the clinical decision with appropriate counselling is highly recommended.

### Strengths and weaknesses of the study

This was a retrospective cohort study with patient reported outcomes, carried out five years after the primary study, which gave ample time for a subsequent pregnancy and hence birth. Although low rates of follow up (40.2%, hence a small sample size) were achieved after five years, limiting the generalizability of our findings, follow up rates were similar for both groups (39.2% for group with previous vacuum extraction and 41.0% for group with previous second stage CS). Recall bias may have been present, especially for mothers that had more than one subsequent birth. A well elaborate questionnaire was used and the questions carefully crafted to reduce recall bias. Although prolonged second stage of labor was the commonest indication for second stage CS among the mothers in their first pregnancy (81%) [7], we acknowledge that some mothers could have had indications that call for repeat CS such as contracted pelvis, which our study did not ascertain.

## Conclusion

Mode of birth in the first pregnancy has important implications on future birth outcomes. Vacuum extraction compared to second stage CS increases a woman’s chance of having a subsequent spontaneous vaginal birth.

### Recommendations

Health professionals need to consider the overall reproductive outcome including mode of birth of future pregnancies for an individual mother. They should continue to offer choice of vacuum extraction/AVB in the second stage of labor for mothers who fulfil its indication since vacuum extraction reduces the risk of a CS in the subsequent birth. Health care professionals need to think twice before they execute a CS, as it carries more risks in the subsequent pregnancies and births. Vacuum extraction is an affordable option so should be well embraced in low resource settings.

## Data Availability

The datasets used and/or analyzed during the study are available from the corresponding author on reasonable request.
